# Improvement in Adenoma Detection Rate with Distal Attachment Device Endo-Wing™-Assisted Colonoscopy: A Randomized Control Trial

**DOI:** 10.3390/diagnostics15091126

**Published:** 2025-04-28

**Authors:** Nabil Mohammad Azmi, Prem Kumar Gopal, Muhammad Irfan Abdul Jalal, Mazian Ismail, Farizal Fadzil

**Affiliations:** 1 Department of Surgery, Faculty of Medicine, The National University of Malaysia, Jalan Yaacob Latif, Bandar Tun Razak, Cheras, Kuala Lumpur 56000, Malaysiamazian83@hotmail.com (M.I.);; 2 UKM Medical Molecular Biology Institute (UMBI), The National University of Malaysia, Jalan Yaacob Latif, Bandar Tun Razak, Cheras, Kuala Lumpur 56000, Malaysia

**Keywords:** adenoma detection rate, Endo-Wing™, colonoscopy, colonic adenoma, colorectal cancer, cancer screening

## Abstract

**Aim:** Endo-Wing™ is a soft silicone device with six wing-like projections attached at the end of the colonoscope that provides superior visualization by flattening the colonic fold and helps to maintain a central view of the colonoscope during withdrawal. This study aims to compare the adenoma detection rate (ADR) between standard colonoscopy and Endo-Wing™-assisted colonoscopy. **Methods:** This is a single-center, single-blind, parallel-group, randomized, actively controlled, exploratory clinical trial conducted between July 2019 and April 2020. Participants aged 45 and above who were symptomatic of colorectal cancer (CRC) or with a history of adenoma and under active surveillance were included. Exclusion criteria included colonic strictures, tumors, active colitis, a previous history of polyposis syndrome, colostomy/ileostomy, or a BPPS score of 0. Participants were subsequently randomized to receive standard colonoscopy (*n* = 96) or Endo-Wing™-assisted colonoscopy (*n* = 96) at a 1:1 ratio using a central block randomization method with varying block sizes. The primary endpoint was the ADR, and the differences between the two groups were evaluated using univariable statistical methods. **Results:** The ADR, the number of adenomas, and the size of adenomas in the Endo-Wing™-assisted colonoscopy group were significantly higher compared to standard colonoscopy (*p* = 0.005, 0.035, and 0.035, respectively). Cecal intubation rates were similar in both groups (*p* > 0.999). The proportions of colonoscopy requiring increased sedation and standard sedation were similar in both groups (*p* = 0.613). No adverse effects of bleeding, perforation, and device dislodgement were reported in both groups. **Conclusions:** This study concludes that Endo-Wing™-assisted colonoscopy improves the ADR compared to standard colonoscopy.

## 1. Introduction

Colorectal cancer (CRC) is the most common and second most common cancer among males and females, respectively, in Malaysia [1]. Globally, it ranks second in terms of cancer-related mortality [2]. Colonoscopy is the gold standard diagnostic modality for CRC screening and diagnosis of other colorectal pathologies. It also possesses a therapeutic role by detecting and removing colorectal adenomas, the precursor lesions to CRC. However, missed lesions remain a key limitation of colonoscopy, contributing to interval colorectal cancer development [3,4,5,6].

The adenoma detection rate (ADR) is defined as the number of adenomas that are successfully detected by colonoscopies, thus serving as the most critical, reproducible, and quantifiable intraprocedural quality indicator [4,5,6]. The ADR reflects the ability of an endoscopist to detect and subsequently remove precancerous adenomas, the primary precursor for CRC. It is hence regarded as a benchmark of endoscopists’ performance, enabling audits and inter-endoscopists or inter-institutional comparisons. If an endoscopist has a low ADR, this indicates the endoscopist’s failure to detect polyps. Corley and co-workers showed that a 1% increase in the ADR reduced CRC risk and CRC-related mortality by 3% and 5%, respectively, thus establishing the importance of ADR as an indicator of successful colonoscopies [5].

The ADR, however, can be affected by various factors such as poor colonoscopy technique, suboptimal bowel preparation, failed cecal intubation, surreptitious adenoma located posterior to the proximal haustral folds, subtle flat lesions, colonic flexure adenomas, and shorter withdrawal time [7]. Hence, the use of enhanced imaging technologies (EITs) such as high-resolution monitors, high-definition colonoscopes, or distal attachment device endoscopy are essential modalities for improving ADR. Nevertheless, the widespread adoption of EITs is limited by its exorbitant costs [8,9]. Therefore, the relatively cheaper add-on endoscopy devices are attractive alternatives for enhancing the ADR.

Hence, we aimed to compare the ADRs between Endo-Wing™-assisted colonoscopy, a new distal attachment device, and standard colonoscopy in our setting. Additionally, we also assessed the cecal intubation rate, differences in polyp distribution between the proximal and distal colon, variations in the number and size of adenomas detected by both modalities, as well as differences in total sedation use and adverse event rates.

## 2. Methods

### 2.1. Study Design and Patient Recruitment

This is a Phase II, single-center, open-label, parallel-group, active-controlled, randomized, exploratory clinical trial conducted at Hospital Canselor Tuanku Muhriz, Faculty of Medicine, the National University of Malaysia (UKM), from July 2019 until April 2020. This study was approved by the National University of Malaysia Ethics Board Committee (Ethics ID: FF-2019-355; Date: 30 August 2019) and adhered to the Declaration of Helsinki and Good Clinical Practice (GCP) guidelines. The reporting of this article adhered to the CONSORT guideline to ensure reporting transparency. This study was funded by the National University of Malaysia from the Medical Faculty Fundamental Research Grant (Grant number: FF-2019-355) and registered in the ClinicalTrials.gov registry (Registration ID: NCT06859125).

### 2.2. Eligibility Criteria

Participants aged 45 and above with symptoms or signs suggestive of colorectal cancer, such as per rectal bleeding, a positive fecal occult blood test, past history of adenoma, positive family history of adenoma or colorectal cancer, altered bowel movement, and chronic constipation, were included in this study. The reason why participants with the latter two symptoms were enrolled is that considering these symptoms merely as functional disturbances carries a significant risk of overlooking structural disorders of the organ. On the other hand, exclusion criteria included those with obstructed colonic tumors, colonic strictures, active colitis, previous colonic surgery for benign and malignant conditions, known polyposis syndrome, colostomy/ileostomy, or poor bowel preparation defined as a Boston Bowel Preparation Scale (BBPS) score of 0.

Colonoscopy was performed by accredited surgeons and postgraduate surgical trainees who have completed their compulsory endoscopic accreditation training. All patients voluntarily consented to study participation and have signed informed consent. The study was conducted in accordance with the Declaration of Helsinki and Good Clinical Practice (GCP) guidelines.

### 2.3. Endo-Wing™ Physical Characteristics

**Figure 1:**
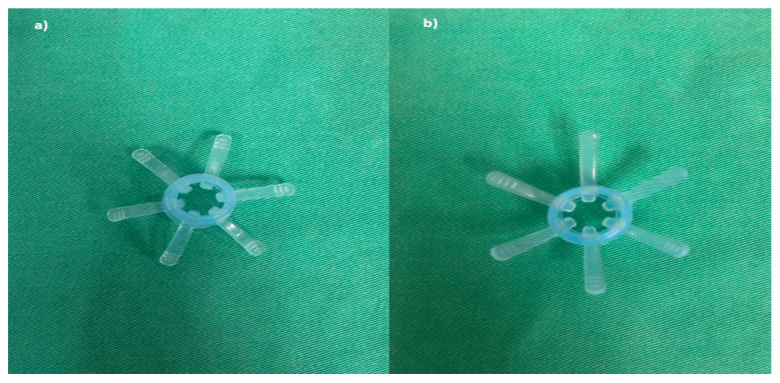
Endo-Wing™ device: Blue Model XT-DL-115AB 10.5–12 mm in size. (**a**) Top view; (**b**) bottom view.

**Figure 2:**
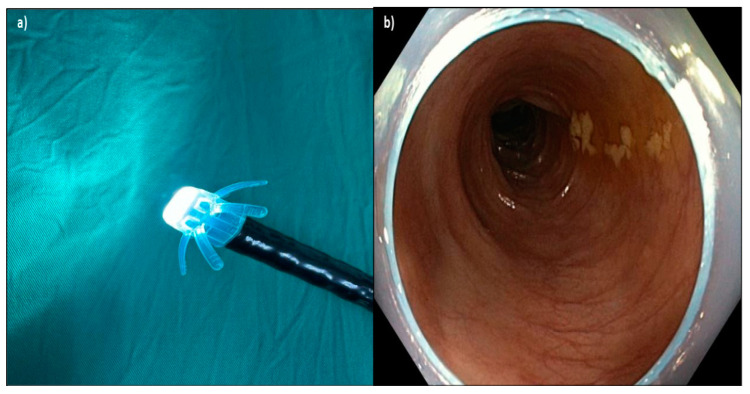
(**a**) Device attached to the end of the colonoscope; (**b**) Endo-Wing™ flattens the colonic folds on withdrawal and keeps the view at the center of the lumen. The distal portion of Endo-Wing™ was observed at the peripheral view, but this neither affected the detection of adenoma nor caused any difficulty in navigating the colonoscope throughout the procedure based on the endoscopists’ feedback.

**Figure 3:**
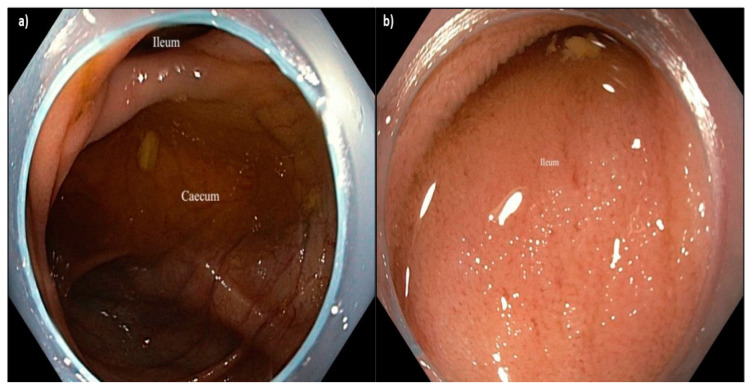
(**a**) Caecum and (**b**) ileal intubation using Endo-WingTM-assisted colonoscopy.Again, the distal portion of the Endo-Wing™ was observed at the peripheral view, but this neither affected the detection of adenoma nor caused any difficulty in navigating the colonoscope throughout the procedure based on the endoscopists’ feedback.

Endo-Wing™ (Shangxian Minimal Invasive Inc, Liaoning, China) is a new, single-used distal attachment device attached at the distal end of standard colonoscopes. It facilitates mucosal inspection by flattening out the mucosal folds and stabilizing the colonoscopes for better mucosal view. It costs only Ringgit Malaysia MYR 45.00 or USD 9.00. It has the unique shape of a soft cap with six wing-like projections and is made of bendable and pliable soft silicone medical-grade material. It was designed to have a single row of rounded wing-like projections, preventing mucosal abrasions observed with other distal attachment devices like the Endo-Cuff. During withdrawal, it flattens and widens the mucosal folds and field of view, enhancing lesion detection and removal (Figure 1, Figure 2 and Figure 3). Nevertheless, no study has yet compared the ADR between Endo-Wing™-assisted colonoscopy and standard colonoscopy.

### 2.4. Sample Size Calculation

The sample size was calculated using PS Software Version 3.1.4.2 (Dupont and Plummer, Vanderbilt University, Nashville, TN, USA, 2018) based on the chi-squared test for independent design. The power (1 − β) and type I error (α) were fixed at 80% and 0.05 (two-sided), respectively. The dropout rate was chosen as 5%, and the control-to-case ratio was fixed at 1:1.

For the values of other parameters required for sample size calculation, a recent study employing colonoscopy adjuncts for visualization enhancement was found to have an adenoma detection rate (ADR) of 45.1% [10]. We hypothesized that standard colonoscopy has an ADR of 28.8%, with a hypothesized difference of 20 percentage points with Endo-Wing™ representing a minimum clinically significant difference (MCID, δ parameter). Using the Pearson chi-squared test, the calculated sample size was found to be 89 patients per colonoscopy arm (n_total_ = 178). After considering a 5% drop-out rate, the final sample size is 94 patients per group (n_total_ = 188 participants).

### 2.5. Randomization, Masking, and Colonoscopy Procedures

The patients were first assessed for study eligibility based on the inclusion and exclusion criteria during pre-colonoscopy surgical clinic visits (two to three weeks prior to colonoscopy) by the medical officers. Eligible and consented patients were subsequently randomly allocated through a central block randomization scheme based on varying block sizes using the randomizer package version 4.1 into either of these two groups: a standard colonoscopy or Endo-Wing™ colonoscopy. Once participants were confirmed as eligible, the medical officers placed a phone call to the statistician to obtain the allocation, ensuring that allocation concealment was preserved. The participants were blinded to treatment assignment, whilst the endoscopists were only masked to the procedural assignment until the commencement of colonoscopy. Other healthcare givers and statistician analyzing the data were not masked to the procedural allotment.

Three liters of polyethylene glycol 4000 electrolyte solution (Fortrans™, Ipsen Pharma) were prescribed and administered in three split doses at 6 pm and 8 pm on the day before the colonoscopic procedure and at 6 am on the day of colonoscopy to ensure adequate bowel preparation. For each dose, one liter of water was mixed with one sachet of Fortrans™. Colonoscopy was then performed in the left lateral position and under monitored sedation according to standard protocol (IV midazolam 3 mg and IV pethidine 25 mg). If the endoscopists deemed the patient was in discomfort or pain, then an additional dose of IV midazolam 2 mg and IV pethidine 25 mg were then administered. Blood pressure, heart rate, and oxygen saturation were monitored throughout the procedure. Bowel preparation quality was assessed using the BBPS system during colonoscopy [11,12] by the performing endoscopists.

All colonoscopies were performed using Olympus Evis Exera III 90 series (Olympus, Tokyo, Japan) or Olympus Evis Lucera 260 series (Olympus, Tokyo, Japan) colonoscopes. If a participant was allotted to the Endo-Wing™ group, the Endo-Wing™ would be attached to the distal tip of the scope, as per the manufacturer’s recommendations. In both groups, the advancement of colonoscopy was performed according to the standard insertion technique. Once the caecum was intubated, the minimum time for withdrawal and examination of the colonic mucosa was fixed at a minimum of 6 min.

### 2.6. Trial Endpoints

The primary outcome of this study is the ADR, which was defined as the proportion of colonoscopies with at least one adenoma detected to the total colonoscopies in that group. Secondary outcomes were the distribution of adenomas by location, number of adenomas detected, size of adenomas, cecal intubation rate, the total amount of sedation used, and adverse effects. Any identified adenomas/polyps were subsequently noted for their size (classified into three groups: 1–5 mm, 6–9 mm, and more than 10 mm), proximal (caecum until transverse colon) or distal (descending colon until rectum) location, and number (group into three categories: 1–2, 3–5, and more than 5 adenomas).

### 2.7. Post-Colonoscopy Procedure Follow-Up and Monitoring for Adverse Events

Patients were reviewed in the endoscopy suite post-colonoscopy for any immediate adverse events (bleeding, perforation) and monitored for at least 1 h at a dedicated recovery bay in the endoscopy suite. Vital signs and oxygen saturation were monitored every 15 min. Prior to discharge, the medical officer in-charge examined the abdomen for signs of tenderness. Once fully conscious, patients were discharged home accompanied by their respective family members or caretakers. All patients were followed up with a telephone call 24 h and one week after for further information on late adverse events.

### 2.8. Statistical Analysis

All analyses were performed using IBM SPSS Version 25 (IBM Corps, New York, NY, USA, 2017). All variables were summarized with mean and standard deviation for continuous variables and frequency and percentage for categorical variables. The normality of continuous variables was evaluated using the Shapiro–Wilk test, Fisher’s coefficient of skewness (normality threshold: ±1.96 for small-to-moderate sized sample), and Q–Q plot. No multiple imputation was carried out for missing data since all variables were completely observed.

Comparison of categorical variables, adenoma detection rate, and adenoma characteristics were analyzed using the exact version of the Pearson chi-squared test (Patel–Mehta algorithm), whilst for continuous variables, the non-parametric Mann–Whitney test was employed. A *p* value of 0.05 (two-sided) was used as the significance threshold, and the estimates of ADR in both groups were reported with their 95% confidence intervals. No subgroup analyses were planned for this trial.

## 3. Results

### 3.1. Patient Recruitment

**Figure 4:**
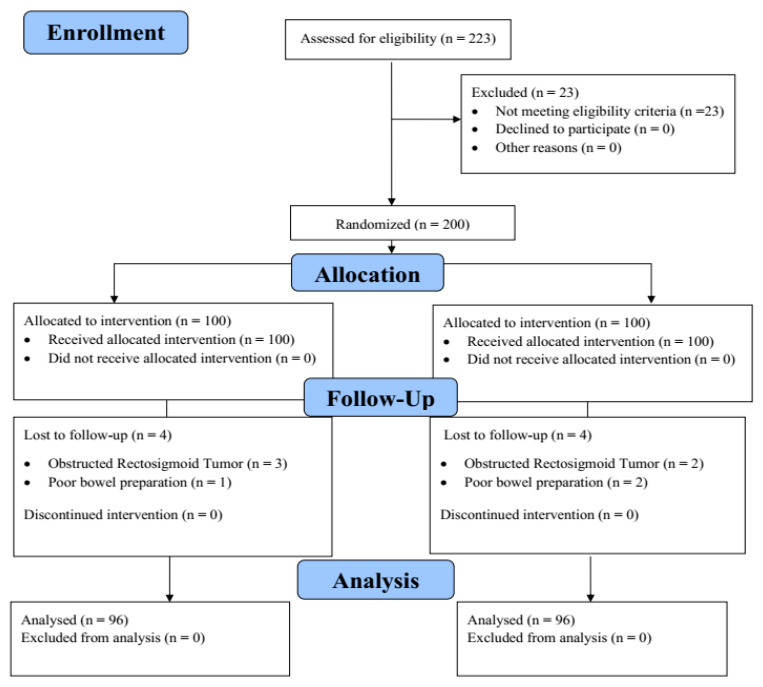
The CONSORT diagram depicting the participant flow throughout the study.

A total of 223 patients indicated for colonoscopy were initially identified in this study. After assessing their study eligibility, 23 patients were excluded due to various reasons (Figure 4). Subsequently, 200 participants were then randomized in equal proportions to both groups, i.e., Endo-Wing™ colonoscopy and standard colonoscopy (*n* = 100 each). However, eight patients (four patients from each colonoscopy group) were excluded from the study due to the following reasons: (a) the Endo-Wing™ group: three patients were excluded for obstructed colon tumor, and one participant was excluded for poor bowel preparation; (b) the standard colonoscopy group: two patients were excluded for obstructed colon tumor, and two patients were excluded for poor bowel preparation. This yields a total of 192 (96 per intervention group) patients for final analysis, which was carried out under the intention-to-treat analytical framework (Figure 4).

Based on Table 1, no significant difference in the baseline age of participants between the standard colonoscopy and Endo-Wing™ groups was observed. In addition, most patients were male (*n* = 111, 57.8%) and the majority of patients had excellent baseline BBPS grades (*n* = 122, 63.5%).

### 3.2. Adenoma Detection Rate and Adenoma Characteristics

Based on Table 2, the ADR in the Endo-Wing™ group was significantly higher than the standard colonoscopy group. In addition, the proportion of a low number of adenoma (1–2 adenomas) detected was significantly higher in the Endo-Wing™ group compared to the standard colonoscopy group (43.8% vs. 25.0%, *p* = 0.035). Furthermore, significantly more smaller-sized adenomas were detected in the Endo-Wing™ colonoscopy compared to the standard colonoscopy group (43.8% vs. 26%, *p* = 0.038). This suggests a possible advantage of Endo-Wing™ colonoscopy over the standard colonoscopic procedure in detecting a small number of adenomas and smaller-sized adenomas.

For adenomas by location, adenomas seen in the proximal part of the colon (standarard colonoscopy vs Endo-Wing^TM^ colonoscopy: caecum 4.2% vs. 11.5%; ascending colon 9.4% vs. 12.5%; descending colon 5.2% vs. 11.5%) were more often identified in the Endo-Wing™ group, but the findings were not statistically significant (*p* = 0.104, *p* = 0.645 and *p* = 0.104, respectively). For the distal part of the colon (the descending and sigmoid colon), the proportions of adenomas detected using standard colonoscopy and Endo-Wing™ were identical (6.3% vs. 6.3%; *p* > 0.999 and 11.5% vs. 11.5%; *p* > 0.999). On the other hand, the proportion of rectal adenomas detected using Endo-Wing™ was higher than the proportion of adenomas detected using conventional colonoscopy; however, the finding was not statistically significant (11.5% vs. 4.2%; *p* = 0.104)

### 3.3. Cecal Intubation Rate, Sedation, and Adverse Effects

In a standard colonoscopy, the successful cecal intubation rate was 95.8%, with only four procedures that failed to reach the caecum. This was mainly due to acute angulation of the colon, difficulty navigating the scope through the hepatic flexure, and colonic looping. In the Endo-Wing™ group, the successful cecal intubation rate was 99.0%, with only one case of failed intubation due to colonic looping.

No significant association was observed between different types of sedation (IV midazolam 3 mg and IV pethidine 25 mg) or added sedation (IV midazolam 2 mg and IV pethidine 25 mg) and different types of colonoscopic procedures (*p* = 0.613). Further information is given in Table 3.

Additionally, no serious adverse events such as bleeding, abrasion, and perforation were observed in these two groups. Moreover, no device failure or dislodgement was seen in the Endo-Wing™ group.

## 4. Discussion

The ADR is an important key indicator for quality colonoscopy, and efforts must be geared toward maintaining and enhancing the ADR. The development of new technologies, including distal attachment devices, is a solution to the challenges of improving ADR during colonoscopy [13]. Experienced endoscopists achieve an ADR as high as 70% [4,5,14]. In this study, we noted that there is a significantly higher ADR in the Endo-Wing™ group compared to standard colonoscopy. With an ADR of 62.8%, it surpassed our a priori expectations and made Endo-Wing™ an enticing distal attachment device for ADR enhancement.

Our result also demonstrated that more small-sized adenomas were detected using this distal attachment device. We postulate that Endo-Wing™ enhances visualization of the colonic folds by flattening them during the withdrawal phase of colonoscopy. The effect of six wing-like projections provided by Endo-Wing™, which fan out during withdrawal, can provide the endoscopist with a superior view. Moreover, Endo-Wing™ keeps the view centered, focusing on the colonic lumen whilst maintaining the colonoscope tip distance to the colon mucosa. These wing-like projections can augment the ADR, as observed by Miyaguchi and co-workers [15]. This distal attachment device did not interfere with the colonoscope’s usual functions, such as water suctioning, washing, and performing diagnostic and therapeutic interventions including polypectomy and biopsy of colorectal tissues.

Regarding safety, no adverse events, such as iatrogenic colonic perforation, tear, or bleeding, were observed during the study. We followed up with all our patients 24 h as well as one week after colonoscopy through telephone calls, enquiring about the occurrence of adverse events such as prolonged alteration of bowel function, pain, and discomfort. The results were encouraging since the patients did not report any adverse events. Hence, our study suggests that Endo-Wing™ is a safe and reliable distal attachment device that does not cause any short-term complications.

It is also worth noting that several endoscopists performing the colonoscopic procedures were postgraduate surgical trainees. However, all of them were accredited by the hospital to perform colonoscopies after undergoing a structured accredited training program and shadowing. Nonetheless, they were still considered less experienced than the specialist surgeons who have years of practice. Nevertheless, they did not report overt difficulties in operating and utilizing Endo-Wing™ during the colonoscopic procedure based on their personal feedback. Therefore, Endo-Wing™ is an enticing distal attachment device that seems to be an invaluable adjunct for improving the ADR among the less experienced surgical trainees performing colonoscopies. Nonetheless, more formal data analyses based on survey-based data collection using a properly designed validated questionnaire are warranted before such a conclusion about the “user-friendliness” of the device can be reliably justified.

Endo-Wing™ provides an attractive option for increasing the ADR due to its cheaper cost compared to more expensive apparatuses such as a narrow-band imaging modality or an artificial-intelligence-assisted colonoscopy device. Thus far, only Miyaguchi and colleagues have investigated the utility of Endo-Wing™ in enhancing the ADR [15] in their trial. They showed a significantly higher average number of adenomas per patient detected by Endo-Wing™-assisted colonoscopy (EAC) than transparent hood-assisted colonoscopy (TAC) (1.13 vs. 0.90 adenomas per patient, *p* = 0.04) [15]. However, they did not find significant differences in adenoma detection rates between EAC and TAC (48.1% vs. 45.0%; *p* = 0.393) [15]. In contrast, we found a much higher ADR in our EAC group, which is 62.8%. This discrepancy could be attributed to differences in baseline risk factors (e.g., older study participants in Miyaguchi et al.’s trial) as well as variations in Endo-Wing™ use (differences in Endo-Wing™ positioning protocol resulting in different effective mucosal exposure), operator skill level, and bowel preparation quality.

Manti et al., in their systematic review and meta-analysis, highlighted that ADRs were higher in colonoscopy assisted with distal attachment devices than standard colonoscopies (45.1% vs. 41.1%; relative risk (RR): 1.18 (95% CI 1.02, 1.37); *p* = 0.03) [9]. Hence, the use of distal attachment devices such as Endo-Wing™ would certainly benefit health centers that have budgetary constraints and, hence, cannot afford to procure a state-of-the-art colonoscopy system with a narrow-band imaging function. As mentioned earlier, there are other distal attachment devices in the market with structural variations, such as Endocuff™ and EndoRing™. Other technologies using retrograde flexion, such as the extra-wide angle colonoscopy systems like the Third Eye Retroscope^®^ System (Avantis Medical System, Sunnyvale, CA, USA) and FUSE Endoscopy^®^ System (EndoChoice, Alpharetta, GA, USA), are also available, but these are significantly costlier [16].

In contrast, other studies using distal attachment devices to increase the ADR have shown varying results [17,18,19]. Biecker et al. observed that the ADR was significantly increased with Endocuff™ compared to standard colonoscopy [20]. On the contrary, another RCT from the Netherlands showed mixed results, and the ADR did not differ significantly between Endocuff and standard colonoscopy [21]. EndoRing, a structurally similar device to Endo-Wing™, showed a significant improvement in ADR compared to standard colonoscopy (49.1% vs. 28.8%; *p* = 0.025) [22]. Our results are thus at least either consistent with previous studies or distinctively superior to certain types of distal attachment devices (e.g., EndoRing) in terms of the ADR. The differences in the ADR of each distal attachment device in different studies are summarized in Table 4. It can thus be concluded that the ADR observed in our trial is comparable to and possibly higher than the ADRs documented in other trials for other distal attachment devices.

In this study, we detected more patients with adenomas of more than 1 cm in size as well as smaller adenomas that are smaller than 5 mm in the Endo-Wing™ than in the standard colonoscopy group. The Endo-Wing™ group also showed a significantly higher percentage of detected single or double adenomas than the standard colonoscopy group. We hypothesize that Endo-Wing™’s superior visualization is the reason for its enhanced ability to detect the readily missed smaller and single adenomas. In the National Polyp Study, increasing adenoma size in those with multiple adenomas was associated with an increased percentage of at least one adenoma with high-grade dysplasia [31]. Adenoma size, especially when greater than 1 cm, is associated with higher grades of dysplasia and a greater risk of adenocarcinoma progress. The number of adenomas detected Is also important, as each adenoma has the potential to become dysplastic and progress to malignant transformation. In addition, interval colorectal cancer incidence is inversely proportional to the number of adenomas detected at colonoscopy [32]. Kaminski et al. have shown that endoscopists with an ADR of less than 11% have a higher chance of missing high-grade adenomas and, therefore, contributing to interval colorectal cancer [6]. Therefore, we believe that Endo-Wing™ can provide a solution to this challenge.

In addition, we also observed that more adenomas were detected on the right side of the colon in the Endo-Wing™ group, with almost more than half of adenomas detected in the caecum, ascending colon, and proximal transverse colon. Based on the literature, right-sided colon adenomas with a size of more than 5 mm tend to be high-risk adenomas compared to left-sided colon adenomas of similar sizes. A right-sided colonic adenoma is frequently missed, is 70% histologically more aggressive, and has a higher propensity to become cancerous [33,34]. With increasing adenoma size, the malignant transformation rate showed a right-sided shift with a significant interaction between adenoma size and right-sided location [35]. Moreover, larger adenomas also tend to be associated with higher odds of invasiveness [35], thus further predisposing the participants to a higher predilection to adenocarcinomatous progression. Again, Endo-Wing™ proves to be an effective tool to enhance the ADR, especially for adenoma detection in the right colon, thus serving as a useful adjunct for enhanced CRC screening or surveillance that may eventually lead to decreased CRC incident cases and CRC-linked deaths.

We also found that the cecal intubation rate was slightly, albeit insignificantly, better in the Endo-Wing™ group (99.0% vs. 95.8%). Four patients in the standard colonoscopy group experienced failed cecal intubation due to excessive looping. In three cases, the colonoscope could not advance beyond the hepatic flexure, while in the remaining case, it reached only the proximal transverse colon. We thus assume that the use of Endo-Wing™ facilitated cecal intubation by helping to center the scope within the colonic lumen, thereby improving visibility, particularly in darker areas, and enhancing the passage and maneuverability of the colonoscope.

In contrast, there is a slightly higher percentage of patients requiring added sedation in the Endo-Wing™ group. The result, however, is not statistically significant. Those affected patients experienced more discomfort rather than pain, which necessitates an additional dose of intravenous midazolam and intravenous pethidine at the discretion of the endoscopists. This discomfort was temporary and resolved completely after the completion of the colonoscopy.

In terms of cost, Endo-Wing™ offers a much cheaper option than other distal attachment devices. In our setting, the per-unit cost for Endo-Wing™ is approximately USD 9.00, which is much less than other alternatives (Endocuff: USD 30 [36]; EndoRings: USD 40; Transparent Cap: USD 25.45). Consequently, Endo-Wing™ is a viable and more cost-effective option compared to other distal attachment device alternatives, particularly in a resource-limited setting with budgetary constraints.

Considering that this device is safe and economical, the Endo-Wing™ distal attachment device is a suitable adjunct for screening colonoscopy of CRC and suitable for use in follow-up colonoscopy in patients with previously diagnosed adenomas, especially right-sided adenomas. Moreover, Endo-Wing™ is also suitable for use by less experienced endoscopists and in a resource-limited setting.

### Study Limitations

Our findings must be cautiously interpreted within the context of several research limitations. First, this is a single-center, randomized control trial that recruited participants around the Klang Valley area, thus limiting the generalizability of our findings. In addition, despite the inclusion of sufficient participants, our sample size may still be inadequate to achieve the minimum statistical power to draw up definitive conclusions by statistically controlling the confounders (e.g., the endoscopists’ years of experience, the level of expertise of the surgical trainee vs. surgeon specialists, elective vs. emergency colonoscopy, etc.) that have a significant influence on the trial outcomes. Hence, to establish more robust evidence, future research should be conducted in a multi-center fashion and recruit a larger number of participants, which will improve the study power and enable the adjustments of confounders using multivariable statistical methods.

Second, we did not compare the withdrawal time between Endo-Wing™-assisted colonoscopy and standard colonoscopy arms. Consequently, the procedural efficiency of these different colonoscopic modalities could not be compared to assess Endo-Wing™’s impact on procedural throughput and its viability for broader clinical adoption. However, Miyaguchi and colleagues demonstrated there was no significant difference in mean withdrawal times between Endo-Wing™-assisted colonoscopy and colonoscopy with transparent hood as the distal attachment device [15], demonstrating Endo-Wing™’s potential procedural efficiency. However, this study compares two different colonoscopy techniques, each using a distinct distal attachment device. Therefore, further research is needed to conduct a direct head-to-head comparison of Endo-Wing™ with standard colonoscopy.

In addition, our study was not designed to analyze missed adenoma rates since it did not employ a tandem colonoscopy design. This design would have provided a more accurate assessment of Endo-Wing™’s effectiveness by enabling per-lesion analysis. Third, our study included patients who have undergone colonic resections, resulting in the reduction of bowel length that might lead to diminished chances for adenomas being detected. Moreover, performing colonoscopies in these patients was more challenging due to bowel adhesions, contributing to the looping problem, which would potentially lessen the cecal intubation rate and increase sedation requirements. Nevertheless, our study showed low rates of failed cecal intubation and added sedation requirements in both colonoscopic groups.

Finally, no histological analysis of the identified adenomas was performed. A better clinical correlation among the size, number, gross morphological features, and histological grades of identified adenomas could have made our results more impactful in terms of the effectiveness of different colonoscopic modalities in detecting high-grade adenomas. Therefore, a future trial that adequately addresses our study limitations will further verify our findings.

## 5. Conclusions

To the best of our knowledge, this represents the first randomized control trial evaluating the efficacy of the Endo-Wing™ distal attachment device in improving ADR. Our single-center study demonstrates that Endo-Wing™-assisted colonoscopy significantly enhances the ADR compared to conventional colonoscopy, exhibiting superior precision in detecting right-sided colonic adenomas and achieving higher cecal intubation rates. As a cost-effective adjunct, this technology holds promise for reducing interval colorectal cancer risk and CRC screening by augmenting the diagnostic efficacy of standard colonoscopy while at the same time maintaining procedural safety. Further research should focus on comparative effectiveness studies, including benchmarking against alternative ADR-enhancing modalities such as narrow-band imaging (NBI) colonoscopy, to better define Endo-Wing™’s role in optimizing ADR.

## Figures and Tables

**Table 1:** The baseline characteristics of study participants (n = 192).

Baseline Characteristics	Total (*n* = 192)	Standard Colonoscopy (*n* = 96)	Endo-Wing™ Colonoscopy (*n* = 96)	*p* Value
Age in years (Median (IQR))	64(14.0)	65.0 (12.0)	63.0 (15.0)	0.922 ^a^
Male sex n (%)	111 (57.8)	58 (60.4)	53 (55.20)	0.559 ^b^
BPSS, excellent score, n (%)	122 (63.5)	65 (67.7)	57 (59.4)	0.235 ^b^

^a^ Based on exact Mann–Whitney test; ^b^ Based on the exact version of the chi-squared test (Mehta–Patel algorithm).

**Table 2:** Associations between different modes of colonoscopy and adenoma detection profiles (*n* = 192).

Characteristics	Standard Colonoscopy (*n* = 96)	Endo-Wing™ Colonoscopy (*n* = 96)	*p* Value ^a^
**Adenoma detection rate; n (%)**	29 (37.2)	49 (62.8)	**0.005**
(95% CI)	(25.1–46.0)	(50.6–70.5)	
**Number of adenoma(s); n (%)**			**0.035**
1–2	24 (25.0)	42 (43.8)	
3–5	5 (5.2)	6 (6.3)	
>5	2 (2.1)	1 (1.4)	
None	65 (67.7)	47 (49.0)	
**Size of adenoma; n (%)**			**0.038**
1–5 mm	25 (26.0)	42 (43.8)	
6–9 mm	3 (3.1)	3 (3.1)	
>10 mm	2 (2.1)	4 (4.2)	
None	66 (68.8)	42 (49.0)	
**Location of adenoma; n (%)**			
Caecum	4 (4.2)	11 (11.5)	0.104
Ascending	9 (9.4)	12 (12.5)	0.645
Descending	5 (5.2)	11(11.5)	0.190
Transverse	6 (6.3)	6 (6.3)	>0.999
Sigmoid	11 (11.5)	11 (11.5)	>0.999
Rectum	4 (4.2)	11 (11.5)	0.104

^a^ Based on the exact version of the chi-squared test (Mehta–Patel algorithm).

**Table 3:** Associations between different modes of colonoscopy procedures and sedation requirement and successful cecal intubation rates (*n* = 192).

Characteristics	Standard Colonoscopy (*n* = 96)	Endo-Wing™ Colonoscopy (*n* = 96)	*p* Value ^a^
**Sedation n (%)**			**0.613**
Standard	89 (92.7)	86 (89.6)	
Added sedation	7 (7.3)	10 (10.4)	
**Successful cecal intubation rate n (%)**	92 (95.8)	95 (99.0)	**0.368**

^a^ Based on the exact version of the chi-squared test (Patel–Mehta algorithm).

**Table 4:** Adenoma detection rate (ADR) for different distal attachment devices in different studies.

Distal Attachment Devices (Manufacturer, Location)	Countries (Sample Size); (Study Design)	ADR (95% CI)	References
Endo-Wing™ (Shangxian Minimal Invasive Inc, Liaoning, China)	Malaysia (192) (RCT)	62.8 (50.6, 70.5)	Our trial
Endo-Wing™ (Shangxian Minimal Invasive Inc, Liaoning, China)	Japan (800) (RCT)	48.1 (43.0, 53.3)	[15]
Endocuff™ (Arc Medical Design, Leeds, UK)	Germany (498); (Two-center RCT)	36.3 (30.2, 42.7)	[20]
Endocuff™™ (Arc Medical Design, Leeds, UK)	Germany (50); (retrospective analysis)	34 (-)	[23]
Endocuff Vision^TIM^ (Arc Medical Design, Leeds, UK)	Multi-national (1772); multi-center RCT	40.9 (37.6, 44.2)	[24]
Endocuff™ (Arc Medical Design, Leeds, UK)	USA (299; Endocuff group) (multi-center RCT)	63.9 (58.1, 69.3)	[25]
Endocuff™™ (Arc Medical Design, Leeds, UK)	Germany (500) (multi-center RCT)	35.4 (29.0, 41.0)	[26]
Endocuff™™™ (Arc Medical Design, Leeds, UK)	Multi-national (8376) (meta-analysis of RCTs)	41.3 (35.7, 47.2)	[27]
EndoRing™ (EndoAid Ltd., Caesarea, Israel)	Netherlands (116) (RCT)	49.1 (35.7, 62.5)	[22]
EndoRing™ (EndoAid Ltd., Caesarea, Israel)	USA (295; EndoRing group); (multi-center RCT)	56.6 (50.7, 62.3)	[25]
EndoRing™ (EndoAid Ltd., Caesarea, Israel)	USA (137; EndoRing group); (cross-sectional)	44 (-)	[28]
EndoRing™ (EndoAid Ltd., Caesarea, Israel)	Multi-national (1257); (meta-analysis of RCTs)	49.1 (42.0, 56.1)	[29]
Transparent cap-assisted colonoscopy (Olympus, Tokyo, Japan) *	Multi-national (2344; cap-assisted colonoscopy group in Figure 5 of reference [30]); (meta-analysis of 7 high-quality RCTs)	38.1 (36.2, 40.2)	[30]

* The manufacturer for the majority of transparent caps used in the individual trial included in the meta-analyses.

## Data Availability

The datasets used and analyzed during the study are available from the corresponding author upon a reasonable request. Public data sharing is not permitted due to the Universiti Kebangsaan Malaysia’s policy that bars public data sharing through data deposition in a publicly available data repository.
